# Remote ischaemic conditioning: defining critical criteria for success—report from the 11th Hatter Cardiovascular Workshop

**DOI:** 10.1007/s00395-022-00947-2

**Published:** 2022-08-15

**Authors:** R. M. Bell, M. Basalay, H. E. Bøtker, S. Beikoghli Kalkhoran, R. D. Carr, J. Cunningham, S. M. Davidson, T. J. England, S. Giesz, A. K. Ghosh, P. Golforoush, A. V. Gourine, D. J. Hausenloy, G. Heusch, B. Ibanez, P. Kleinbongard, S. Lecour, K. Lukhna, M. Ntsekhe, M. Ovize, A. D. Salama, G. Vilahur, J. M. Walker, D. M. Yellon

**Affiliations:** 1 The Hatter Cardiovascular Institute, University College London, 67 Chenies Mews, London, WC1E 6HX UK; 2 Aarhus University Hospital and Aarhus University, Aarhus, Denmark; 3 Royal Free Hospital, London, UK; 4 Stroke, Division of Mental Health and Clinical Neurosciences, School of Medicine, University of Nottingham, Nottingham, UK; 5 Centre for Cardiovascular and Metabolic Neuroscience, Neuroscience, Physiology and Pharmacology, University College London, London, UK; 6 CVMD, Duke-NUS, Singapore, Singapore; 7 National Heart Research Institute Singapore, National Heart Centre, Singapore, Singapore; 8 Cardiovascular Research Center, College of Medical and Health Sciences, Asia University, Taichung City, Taiwan; 9 Institute for Pathophysiology, West German Heart and Vascular Center, University of Duisburg-Essen, Duisburg, Germany; 10 Centro Nacional de Investigaciones Cardiovasculares (CNIC), IIS-Fundación Jiménez Díaz University Hospital & CIBERCV, Madrid, Spain; 11 CIBER de Enfermedades Cardiovasculares (CIBERCV), Madrid, Spain; 12 IIS-Fundación Jiménez Díaz Hospital, Madrid, Spain; 13 University of Cape Town, Cape Town, South Africa; 14 INSERM U1060, CarMeN Laboratory, Université de Lyon, Groupement Hospitalier Est, Bâtiment B13, F-69500 Bron, France; 15 Institut de Recerca de l’Hospital de la Santa Creu i Sant Pau, CIBERCV, Barcelona, Spain

**Keywords:** Ischaemia reperfusion injury, Remote ischaemic conditioning, Cardiovascular

## Abstract

The Hatter Cardiovascular Institute biennial workshop, originally scheduled for April 2020 but postponed for 2 years due to the Covid pandemic, was organised to debate and discuss the future of Remote Ischaemic Conditioning (RIC). This evolved from the large multicentre CONDI-2–ERIC–PPCI outcome study which demonstrated no additional benefit when using RIC in the setting of ST-elevation myocardial infarction (STEMI). The workshop discussed how conditioning has led to a significant and fundamental understanding of the mechanisms preventing cell death following ischaemia and reperfusion, and the key target cyto-protective pathways recruited by protective interventions, such as RIC. However, the obvious need to translate this protection to the clinical setting has not materialised largely due to the disconnect between preclinical and clinical studies. Discussion points included how to adapt preclinical animal studies to mirror the patient presenting with an acute myocardial infarction, as well as how to refine patient selection in clinical studies to account for co-morbidities and ongoing therapy. These latter scenarios can modify cytoprotective signalling and need to be taken into account to allow for a more robust outcome when powered appropriately. The workshop also discussed the potential for RIC in other disease settings including ischaemic stroke, cardio-oncology and COVID-19. The workshop, therefore, put forward specific classifications which could help identify so-called responders vs. non-responders in both the preclinical and clinical settings.

## Background

Remote Ischaemic Conditioning (RIC) has been shown consistently to be an effective experimental intervention for the reduction of ischaemia–reperfusion injury in all organ systems and animal species that have been studied to date. The phenomenon is not new. Przyklenk’s group first described in the heart that non-injurious ischaemia in one coronary artery territory (i.e., circumflex) would lead to protection in an adjacent coronary vascular bed (distal to the left anterior descending occlusion in this study) almost 30 years ago [78]. MacAllister’s group were the first to demonstrate inter-organ RIC in humans [55] and this observation has since been extended to reveal that practically any organ system put under ischaemic stress leads to the upregulation of cytoprotective pathways in remote visceral tissues [46, 57]. These prosurvival pathways have been shown repeatedly to reduce cell death and infarction in response to injurious ischaemic injury in models that replicate clinical syndromes, such as acute myocardial infarction, acute ischaemic stroke and acute ischaemic kidney injury [20, 31]. Given the promise of strong basic research, and indeed the promising data from clinical proof-of-concept trials, there has been a strong desire to translate what had promised to be a robust, low-cost, non-pharmacological intervention to further improve patient outcomes.

## Failure of translation

However, attempts at clinical translation thus far have not realised the uniformly effective intervention that had been either anticipated or hoped for. In recent years, large patient outcome trials looking at the efficacy of RIC in the context of primary percutaneous intervention and cardiac surgery have proven predominantly neutral [43]. The same is true in the setting of renal transplantation, although the results are somewhat more mixed. While most trials in deceased donor transplantation showed no benefit following RIC [59, 97], in some—but not all—living-donor studies of efficacy, estimated glomerular filtration rate (GFR) was improved in those patients who received RIC [1, 71, 91]. The critical question, therefore, is “Why?” Why is there an apparent disconnect between positive animal models and neutral clinical trials? This vexing clinical translational question has been the subject of previous discussions and debate [7, 20, 28, 42, 43, 49, 83] that have led to a number of recommendations and actions, but the question of the viability of RIC as a clinical intervention in acute ischaemic emergencies remains. Is RIC simply an intervention that cannot be translated from animal studies into the clinical setting (despite encouraging data from early phase clinical studies), or are we looking at the wrong target for intervention? Despite these doubts, grant awarding bodies are still funding clinical trials. This speaks volumes regarding the persisting clinical need to improve outcomes following ischaemic emergencies and the acknowledgement of the relevance of reperfusion injury. However, is the continued study of RIC an appropriate and ethical use of resources? This was the basic premise for the 11th Hatter Cardiovascular Research Workshop, recently held in South Africa. The latest research in RIC in various models, including heart, kidney and the brain were discussed and debated and the question of how best to target RIC, an intervention that has been repeatedly demonstrated to be safe in the clinical setting, addressed. A recurring theme arising in the discussions was on whether it was time to “get back to basics” and to finally identify the complete and precise mechanism by which RIC works, or whether RIC is a “therapy in need of re-targeting”. To this end, it may be useful to define which are the critical criteria for successful induction of cardioprotection using RIC—a point that will be elaborated below.

## Back to basics: understanding remote ischaemic conditioning

While gaps in knowledge belying RIC signalling were not felt to represent a significant obstacle to clinical translation, recent neutral clinical outcome trials will force the field to redress these omissions.

### How does remote ischaemic conditioning work?

In the ~ 30 years since the first description of RIC, the field has expanded significantly, leading to a far greater understanding of both the mechanisms preventing cell death following injurious ischaemia and subsequent reperfusion, and the key target cyto-protective pathways recruited by protective interventions, such as RIC. However, there remain significant gaps in our scientific knowledge as to how RIC recruits signalling from one tissue bed, such as a limb, to result in protection in remote organs. This knowledge gap in how RIC functions could be critical, since it could have led to vulnerabilities of this pathway being overlooked. For example, the potential for the interaction of co-morbidities and coincident medical interventions to interfere with the effective recruitment of cytoprotective signalling [26, 56]. Another outstanding question relates to whether RIC can potentially be used to limit ischaemic injury itself as opposed to reperfusion injury alone [6, 56].

**Fig. 1:**
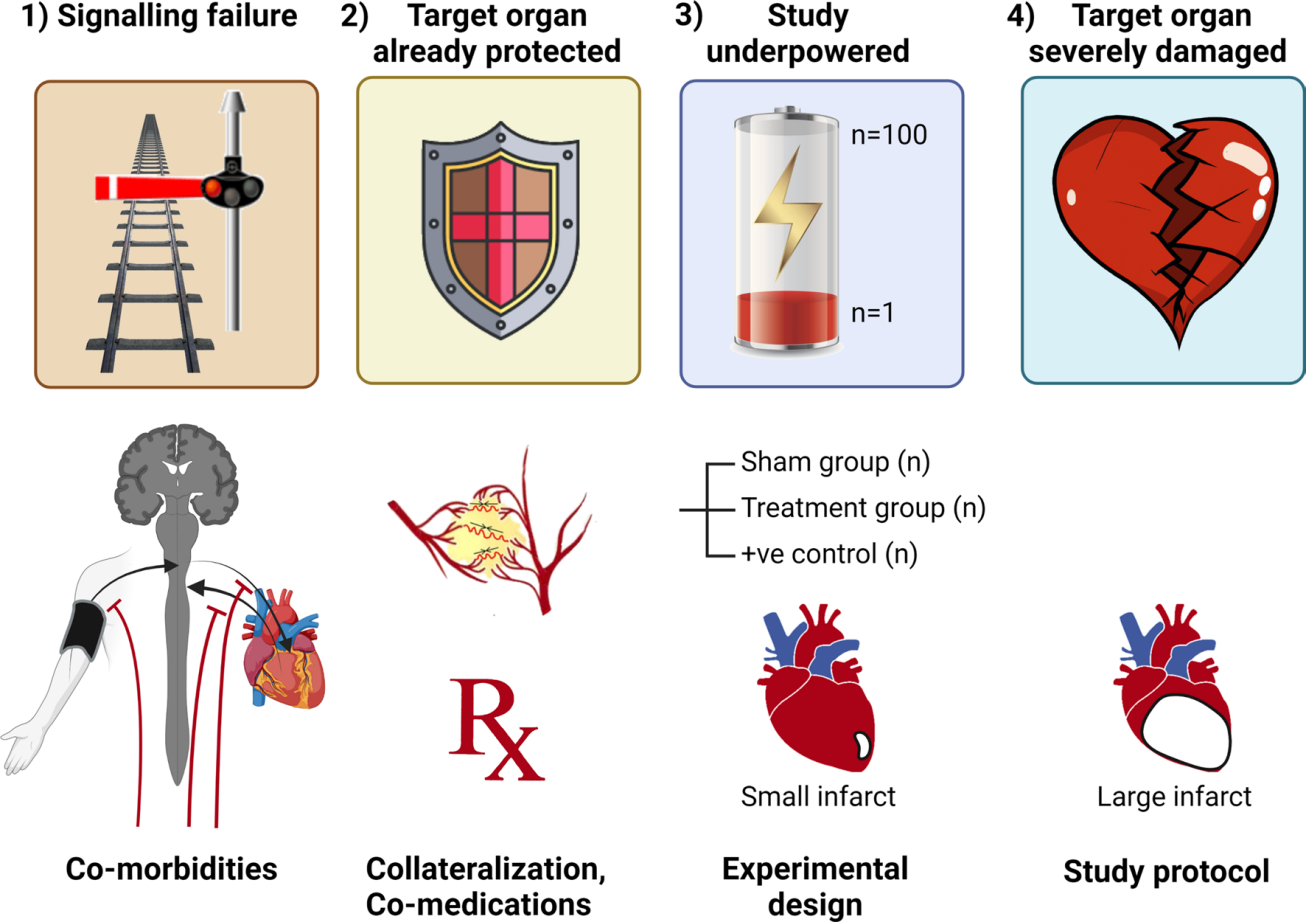
Four main categories of potential failure in the application of Remote Ischaemic Conditioning

The current model of RIC signalling from the limb to a remote internal organ suggest that this is via a neuro-humoral pathway (Fig. 1) [3, 57]. The neural pathway recruits afferent innervation of the peripheral tissue undergoing RIC [75]. These sensory nerves are activated by the ischaemic stimulus, and recruit efferent activity of the vagus nerve [68, 73]. Vagotomy at various levels leads to abrogation of cardioprotection induced by RIC [76]. Gourine’s group found that RIC is abolished in the rat model by total subdiaphragmatic vagotomy, gastric vagotomy or sectioning of the posterior gastric branch. However, the efficacy of RIC cardioprotection was unaffected by hepatic, celiac or anterior gastric vagal branch transection [67, 69]. Moreover, electrical stimulation of the posterior gastric branch of the vagus reduced infarct size, mimicking the effect of RIC [67]. Heusch’s group similarly found that vagotomy abrogated RIC, although in the pig, in contrast to the rat, splenic rather than posterior gastric vagotomy abrogated RIC-induced protection [64]. In fact, in pigs the spleen is the central relay organ between the neuronal and humoral cardioprotective signalling pathways [44]. However, the nervous system involvement does not appear to be limited to the direct effect of the vagal innervation on the myocardium. Instead, remote organ protection appears to require the release of a humoral factor (which could be under the nervous control). This was first demonstrated by Redington’s group through the extraction of plasma from conditioned subjects and perfusing this through a naïve heart, which demonstrated a significant reduction in infarct size in the naïve heart as it did in the heart of the animal that directly received RIC [86]. Yellon’s group confirmed this and further demonstrated the co-dependence of the neural and humoral pathways in the mechanism of RIC [75]. However, the humoral component and its source remain controversial, although it is thought to be a small molecule (of less than 12–14 kDa) [75]. Numerous potential candidates have been suggested, e.g., the 3.3 kDa Glucagon-like peptide-1 (GLP-1) [4, 72] and the 18 kDa immunoregulatory glycoprotein, interleukin-1α, interleukin-10, Stromal-derived factor 1a, and nitrite [13, 22, 80] (reviewed in [57, 90]). Protection in circulating blood, however, is not only mediated by circulating soluble factors [19], but also by components mediated by blood cells (e.g., platelets [79] and extracellular vesicles [92]), which may mask relevant protective components intracellularly or intravesicularly. As yet, however, there is not a definitive answer to the elusive question of the circulating factor, particularly in man [50, 65], and yet, if this could be resolved, then we would be in a better position to develop a biomarker of RIC signalling activation, to facilitate our understanding the impact of co-morbidity upon RIC and how such problems in clinical translation could be overcome.

### Are we giving the correct remote ischaemic conditioning stimulus?

Perhaps surprisingly, the duration of limb ischaemia, the number of cycles required and even the pressure of the cuff used to occlude arterial blood supply to the limb have received limited attention [53, 81, 93]. The regimen that has been most widely deployed to date—that of three to four cycles of 5-min ischaemia, using a standard blood pressure cuff that is generally inflated to 200 mmHg (or alternatively 20 mmHg above systolic blood pressure), appears to have been arrived at almost empirically, although there are abundant studies that show this protocol to be effective in a variety of animal models. However, is this protocol best optimised for effectiveness in multi-morbid patients with a background polypharmacy?

Perhaps the closest we can currently come to addressing this question is to look at the data that have already been published in the setting of both acute myocardial infarction and ischaemic stroke. Several groups have undertaken a meta-analysis of remote conditioning protocols in basic and clinical settings, with the aim of ascertaining their ability to protect and whether any observation can be made with respect to the ischaemic stimulus itself in these two disease settings [12, 49, 62, 94]. In the stroke model [94], the meta-regression did not reveal any relationship between cycle number or cycle duration, the number of limbs used simultaneously in the application of RIC and the efficacy of RIC to reduce cerebral ischaemic injury. RIC must be applied prior to or possibly shortly after the onset of reperfusion, since its efficacy rapidly diminishes the later it is applied after the onset of myocardial reperfusion [2]. In terms of the “dose” of RIC (the total duration of limb ischaemia applied), as long as the ischaemic conditioning durations were between 15 and 45 min (irrespective of cycle number used to achieve this), cerebral infarct volume was demonstrably reduced. However, it was not possible to assess the interaction of age, gender or co-morbidity. In the myocardial infarct model, there was also no association observed between various cycle lengths, with 10-min cycles being equally effective as 5-min cycles. As has been discussed at previous meetings and in numerous reviews, any one of these factors, and possibly all three, are important in considering the efficacy of intervention [26, 37, 96].

Yellon’s group showed that in some cases, co-morbidities can be overcome through increasing the number of cycles of RIC (effectively increasing the “dose” of the conditioning stimulus) to overcome a weakening of fundamental cyto-protective signalling cascade response to standard RIC protocols in animal models, but multiple co-morbidities or indeed co-medications are rarely, if ever, fully investigated in animal models. In reality, the true model that we need to consider here is the human “model”, more specifically the patient who commonly presents with an acute ischaemic syndrome, with all the attendant co-morbidities, such as atherosclerosis, age, diabetes etc., that these patients inevitably present with. In retrospective analyses of the limited existing clinical data, there is less solid clinical evidence for an effect of co-morbidities and co-medications on cardioprotection. Evidence for an influence of co-medications comes from studies using antiplatelet agents which can, in themselves, elicit cardioprotection thus limiting the potential for further protection [15, 98]. In addition, the anaesthetic, propofol has also been shown to abrogate RICs protection during cardiovascular surgery [56].

In terms of optimising RIC “dose” in humans, currently the only way a dose–response curve can currently be constructed is through assessment of a clinical endpoint, such as infarct size as the biomarker of RIC efficacy. This is a difficult and expensive way of optimising a RIC regimen that may require many iterations in many different populations of patients with differing co-morbidities to identify the ideal RIC protocol. It is possible that flow-mediated dilatation or heart-rate response could be used as a biomarker of an effective RIC application [55], but bedside acquisition of such measurements requires considerable skill to perform well, with results open to interpretation bias. A blood biomarker would be preferable for the ease and consistency of measurement—and perhaps a panel of biomarkers to enable us to not only demonstrate an activated vagal signalling arc, but also release of humoral mediator and downstream cytoprotective activity within the organ of interest itself. The perfused rodent heart has been used as a bioassay for humoral cardioprotective factors released in human individuals undergoing RIC [50, 65].

### Classifying failure of remote ischaemic conditioning protection

It would be very helpful to be able to identify the reasons for failure to protect against injurious ischaemia by RIC, and thereby identify likely responders and non-responders. As alluded to previously, patients are frequently multi-morbid and are often on therapy that can modify cytoprotective signalling.

As discussed above, the timing and dose of the RIC intervention is important for obtaining maximal cardioprotection. As such, inappropriate timing or insufficient dose may result in failure of RIC (Category 1a RIC protection failure, Table 1). Classical cardiovascular risk factors, such as diabetes, are well known to reduce neuronal signalling—the neuropathy encountered in diabetes includes sensory nerve dysfunction and vagal autonomic dysfunction [52]—and this may well result in failure of RIC (Category 1b&c, respectively, Table 1). Even non-traditional co-morbidities, such as chronic renal impairment, leads to vagal dysfunction [84] that in turn is likely to result in category 1b or 1c RIC protection failure. Co-morbidities may impair release of the humoral factor or result in the target organ not responding to the humoral factor, again resulting in failure of RIC (categories 1d and 1e, respectively, Table 1).

**Table 1:** Defining critical criteria and potential pitfalls for success

	Category	Possible causes
1	Signalling failure (Co-morbidities)	(a) Insufficient dose of RIC or inappropriate timing to initiate signalling (b) Failure of nociception afferent signalling (c) Failure of efferent vagal signalling (d) Failure of sufficient release of humoral factor (e) Failure of target organ to respond to humoral factor
2	Target organ already protected prior to RIC (e.g., Co-medications)	(a) Presence of significant collateral flow reducing infarct development (b) Administration of co-medications that are themselves cardioprotective (morphine, P2Y_12_ inhibitors, propofol, statins etc.)
3	Study underpowered (Experimental design)	(a) Duration of ischaemia insufficient to lead to a large enough infarct to detect a significant change in infarct size (b) Insufficient numbers per group to detect a significant difference
4	Target organ severely damaged (Study protocol design)	Duration of ischaemia excessive, resulting in irreversible injury to the majority of the area at risk

Possible explanations for the failure of RIC to demonstrate cardioprotection

The drugs used to manage co-morbidities (for example, HMG CoA reductase inhibitors for hypercholesterolaemia) or the acute ischaemic syndrome itself (e.g., the use of P2Y_12_ receptor antagonists in acute ST-elevation myocardial infarction (STEMI)) are frequently found themselves to be cardioprotective. We have previously discussed the so called “success hypothesis” [7], whereby the success of optimal medical management in cardiac ischaemia management in reducing mortality from acute coronary syndromes is likely to cause coincident and largely unintended up-regulation of the same cyto-protective pathways that are utilised by RIC. In other words, we are already pharmacologically conditioning our patients (Category 2b). If the aforementioned pharmacological cytoprotection is realised via the same pathway as RIC, then RIC will not further up-regulate this protection. This has been demonstrated by Yellons’ group in a simple study in which rats were given a similar cocktail of medications that are routinely administered to patients presenting with a STEMI. The combination of an opiate, a P2Y_12_ inhibitor, and heparin themselves reduced infarct size equivalent to that seen following RIC alone, and RIC was not able to add any further benefit to the drug combination. In such a scenario, the RIC cytoprotective signalling is thus likely “to have reached a ceiling by the concomitant drug therapy to which RIC itself could not further augment [36]—a Category 2b RIC protection failure (Table 1).

This may have been the case in the recent large multicentre CONDI-2–ERIC–PPCI study which demonstrated that RIC was not able to improve clinical outcomes [33]. What is more, it is interesting to note that the mortality from cardiovascular death in the control group was only 2.7%. This suggests that these clinical studies using patients in countries with well-established cardiac coronary care, are of such low risk that additional therapy is unlikely to demonstrate any further benefit in outcome. This is despite the fact that, *outside of clinical studies*, mortality rates for hospitalized STEMI patients in Europe remain in the region of 15–25% [38], which demonstrates that there clearly remains a need for cardioprotective strategies, even if it is difficult to demonstrate in this population. A solution to this quandary may be to undertake future studies using this low-cost intervention in developing countries, where presenting patients are at significantly higher risk [10, 35, 47].

Ovize’s group undertook an extensive, retrospective analysis of their CIRCUS study, which was a phase 3 study designed to investigate the protective effect of cyclosporine, a cyclophilin-D inhibitor. In pre-clinical animal and early clinical proof-of-concept studies, cyclosporine had shown great promise in reducing infarct size through inhibition of the formation of the mitochondrial permeability transition pore [30, 34, 77, 87]. Nevertheless, the CIRCUS study was ultimately neutral [18]. Due to short pain-to-balloon times (i.e., the time between onset of symptoms and the percutaneous intervention to re-open the occluded vessel), infarct sizes were relatively small. This would imply a Category 3a RIC protection failure (Table 1). Nevertheless, it was infarct size that appeared to have the greatest impact on the ability to see a cytoprotective signal [9]. Interestingly, in their analysis, hypertension, renal dysfunction, post-PCI TIMI flow grade and treatment with beta-blockers or ACEI all had a major influence on clinical outcomes *without* significantly affecting infarct size or adverse LV remodelling [9], which led the authors to suggest that infarct size alone maybe poor surrogate for outcome. On the other hand, infarct size has been correlated with outcome in large STEMI studies [88]. Interestingly the relationship between infarct size and long-term outcome has not to our knowledge been investigated in animal studies [39].

Heusch and Kleinbongard emphasised that not only infarct size but also coronary microvascular obstruction is an important but possibly neglected endpoint of cardioprotection [32, 40, 41, 70]), which carries prognostic information for patients´ outcome [23, 24].

### Optimising the design of future clinical trials

Given the multiple potential points of failure in the RIC mechanism of protection highlighted in Table 1, there are numerous ways in which clinical trial design can go awry. Similarly, many of these issues are not reflected in the design of fundamental science-based studies. This begs an important question: do we need more complex animal models to mimic clinically relevant models of disease and patient management, or should clinical trials be more reductive, applying rigorous eligibility criteria, to select only those patients who closely reflect the animal models? The latter is likely to be the most useful in establishing proof-of-efficacy, although there is a role for the former to gain a more comprehensive understanding of the pitfalls of implementing RIC in the clinical setting. Gourine suggested that a possible means of stratifying or identifying responders would be to identify patients with preserved vagal autonomic function using heart rate variability analysis or cardiopulmonary exercise testing [45]. Heusch emphasized that vagal function is indeed central to protection by RIC [45].

In retrospect, the large outcome studies performed thus far have not been designed to detect a reduction in I/R injury. In all cases, studies have been designed to optimise recruitment through inclusion of all-comers. In most studies of acute myocardial infarction, there was no stipulation of minimum duration of chest pain, the site of coronary occlusion, the presence of collateralisation, patient age, whether patients were previously naïve of medical therapies and they only documented the presence of co-morbidities. Thus, cardioprotection studies have tended to recruit older, multi-co-morbid patients (thus with a high likelihood of a Category 1 RIC protection failure); with short symptom-to-balloon times; distal, non-LAD, TIMI > 1 lesions; possibly with collateralisations (high likelihood of Category 3 failure); and given full optimal medical therapy (high likelihood of Category 2 failure). Another factor may be that approximately 1/3 STEMI cases are caused by plaque erosion, which typically results in smaller infarctions and fewer complications in the microvasculature [58]. While these limitations may not have fully masked a positive signal, detecting that positive signal within the background “noise” of the study becomes significantly harder and skews the assumptions of the original power calculations. Ibanez raised the possibility of using an adaptive study design for clinical cardioprotection studies to optimise and improve the robustness of clinical trials [16].

However, through better understanding of the modes of RIC failure, it should be possible to construct a clinical trial that is not, from the outset, biased towards neutrality through inappropriate patient selection. Both the Remote Ischaemic Conditioning in STEMI Patients in Sub-Saharan AFRICA (RIC–AFRICA) Study (NCT04813159) and the RIP–high study offers an opportunity to address this important distinction by designing a study to intervene in patients who are at high risk. In the former study, the majority of patients are naïve to the medical therapies that are innately cardioprotective—thus potentially avoiding a category 2b RIC protection failure.

Yellons’ group revealed that in their proof-of-concept Mauritian ERIC–LYSIS Study (NCT02197117, a positive signal towards cardioprotection was seen, with a 32% reduction in area-under-the-curve troponin-T release [99]. This study gave some cautious optimism that in a high-risk group of patients, in which thrombolysis (not PPCI) was used, and in a patient group which had significant levels of diabetes and other co-morbidities and given less co-medication, there may yet be a need to manage patients throughout the world in health care systems with limited resources. Therefore, using a cheap and low risk intervention such as RIC may yet prove beneficial. This will be tested in patients recruited to the RIC Africa study who will be high risk, often with signs of heart failure (Killip > 1), are very likely to present much later than found in well-developed medical systems in the Western world [66]. This will avoid a Category 3 RIC protection failure, but such an approach carries with it a risk of a Category 4 RIC protection failure, as these patients may present very late owing to long transit times to their local heart attack centre, and thus more likely to present with a fully infarcted risk zone. In addition, thrombolysis rather than primary percutaneous intervention is the revascularisation modality of choice, and thus the timing and even success of re-canalisation of the culprit vessel will be unknown, but this was also the case in the ERIC–LYSIS study. That said, there may be benefits even in those patients, where vascular patency is achieved too late to salvage the ischaemic myocardium, albeit in preventing adverse remodelling and arrhythmia.

The RIP–HIGH trial will also examine this hypothesis of whether RIC can protect in a high-risk patient group. RIP–HIGH is a two-arm randomized controlled trial with an adaptive study design aiming to compare the impact of combined remote ischemic conditioning and local ischemic postconditioning vs. standard of care on clinical outcome in high-risk STEMI patients undergoing primary percutaneous coronary intervention (NCT04844931).

Another population where RIC may be ideally suited is in acute ischaemic stroke [5]. Substantial strides have been made in recent decades in the management of stroke, particularly with thrombolysis that has recently been further augmented by interventional radiology and arterial thrombectomy. While patients will typically have co-morbidities, in contrast to the cardiac field, P2Y_12_ inhibitors, heparin and opiates are not routinely used, and statin usage for primary prevention is relatively less commonly applied—thus potentially avoiding a Category 2b RIC conditioning failure. In this context, Remote Ischemic Conditioning in Patients With Acute Stroke Trial (RESIST)(NCT03481777) [8] and Remote Ischaemic Conditioning After Stroke 3 (RECAST-3, ISRCTN63231313) led by England’s group, are aiming to study RIC in the context of ischaemic stroke, including sub-studies that aims to investigate RIC in the context of mechanical thrombectomy. RECAST-3 builds on the success of RECAST and RECAST-2, both providing positive biomarker signals of RIC efficacy [25], and builds on the positive preclinical animal model which has shown a reduced cerebral infarction with RIC [3].

Other organ systems could be similarly targeted, for which ischaemic emergencies are encountered or transient ischaemia can be anticipated (for example in surgery and particularly in the handling of donor organs)—and it could be argued that it may be worth revisiting certain studies such as the REPAIR Study (REmote preconditioning for Protection Against Ischaemia–Reperfusion in renal transplantation) [91] in the light of our improved understanding of how best to design appropriate studies avoiding the categories seen in Table 1.

### Novel targets for intervention

While the focus of large clinical trials to date has appropriately been on ischaemia and reperfusion injury, there are other emerging fields in which RIC may potentially find a role. Discussion in this workshop covered a range of novel targets that may be amenable to RIC intervention. In this regard, Redington’s group recently demonstrated the ability of RIC to improve cardiac output and organ function in the context of lipopolysaccharide-induced sepsis [51]. Optimising clinical outcomes in severely ill patients on the intensive care unit has been a holy grail for intensivists, and other groups have also seen similarly positive signs of positive outcomes. Whether RIC can prove to be a helpful intervention in the sedated, ventilated patient in all forms of sepsis, be it bacterial, viral or fungal, in in the management of shock, remains to be seen.

Sepsis is associated with an increase in cytokines similar to that observed in patients diagnosed with COVID-19 [74]. This particular question is being investigated in a clinical study designed to ascertain whether RIC can reduce cytokine levels and prevent deterioration to critical care in patients with COVID-19, and we await the outcome of the clinical studies [21].

Another area discussed, which is becoming increasingly relevant to cardiologists and oncologists alike, is the impact of cancer therapy (particularly chemotherapeutic agents, such as the prototypical anthracycline) upon cardiac injury and the risk for the late emergence of heart failure and iatrogenic cardiomyopathy. It is certainly clear that repeated cycles of anthracyclines lead to an accumulative injury to the heart, often realised by the release of troponin (e.g., high-sensitivity cardiac Troponin-T [hs-TnT]). Of note, in some cases anthracycline cardiotoxicity results in cardiac contractile impairment without death of cardiomyocytes. These cases might not be picked up by hs-Tn evaluation, and more sensitive modalities, such as cardiac magnetic resonance imaging or strain echocardiography should also be considered. In fact, in some ways, anthracycline cardiotoxicity may be a more suitable target for RIC than STEMI, since the timing for the intervention of RIC is more convenient (i.e., it can be planned to occur immediately before the exposure to the toxic agent: anthracycline infusion), there is no issue regarding the area at risk or coronary collateralization extent. This has inspired studies of RIC in the setting of anthracycline cardiotoxicity [14, 20, 29], to which we await the outcome with interest. The REmote iSchemic condItioning in Lymphoma PatIents REceiving ANthraCyclinEs (RESILIENCE) trial, NCT05223413, was discussed in detail. This is an EU-funded project that will enrol 600 patients at risk for anthracycline-induced cardiotoxicity and randomize them to weekly RIC or standard of care with a comprehensive, serial, cardiac magnetic resonance imaging evaluation.

However, the mechanism of anthracycline-mediated myocardial injury remains unclear, and how chemotherapy may potentially impact the generation of the RIC signal has also not been described [48, 89]. Moreover, hs-TnT may not be the ideal biomarker, as cardiac troponin T can be expressed by non-cardiac muscle [82, 85, 95]. It was suggested that other markers of cardiac injury should, therefore, be considered, such as cardiac myosin-C (c-MyC) [54], and the novel biomarker of ischaemic injury, glycosylated apolipoprotein J [17] may provide further sensitive and specific markers of myocardial injury.

## Conclusions and the path forward

The workshop participants concluded that, although some aspects of RIC remain an enigma it is recognised as a powerful cytoprotective intervention in experimental models. Furthermore, it has led to remarkable advances in our understanding of the mechanisms and progression of cell death following lethal ischaemia and reperfusion and has provided novel insights into neuro-humoral signalling that appears to link different organ systems under conditions of stress. However, thus far at least, it has failed to translate into an effective clinical therapy.

Applying RIC in modern clinical practice has proven considerably more difficult than expected—the reasons for its failure have been widely discussed. One conclusion of those attending this workshop was the importance to return to basics: to fully define the whole RIC cyto-protective signalling arc and to understand how co-morbidities and concomitant medical therapies may interfere with RIC signalling and the downstream cytoprotective signalling in the organ itself. On that basis, we propose a simple scheme for breaking down and understanding how RIC protection signalling may be inhibited, and based on this understanding, ensure that future clinical trials are cognisant of these potential issues and patient inclusion/exclusion criteria are developed to ensure that trials can be appropriately powered to provide a definitive answers to the question: is RIC a potentially useful clinical intervention, or should it remain an incredibly useful research tool in which to expand our knowledge of ischaemia and reperfusion injury.

It is also clear that we need a fuller understanding of the neuro-humoral signalling pathway and need for biomarkers to measure activation of the RIC signalling pathway, so that conditioning protocols can be optimised and potentially identify patients who are RIC responders, and those who are not.

In pre-clinical studies, there are already excellent guidelines for the design and reporting of fundamental science studies, such as those advised in neuroprotection: the Stroke Treatment Academic Industry Roundtable (STAIR) guidelines [27], the 20/20 standards for translational stroke research [60] and RIGOR guidelines [61]. Similar guidelines, called the IMproving Preclinical Assessment of Cardioprotective Therapies (IMPACT) criteria, have recently been proposed for studies of cardioprotection [63]. Such approaches are highly desirable from the perspective of transparency and reproducibility [11].

The workshop participants debated whether pre-clinical studies should be made more complex so as to more accurately reflect the clinical scenario when patients present with an acute ischaemic syndrome. There is certainly a place for such studies, but there is also an argument that increasing complexity of basic studies moves away from discovery into something else entirely, and as part of the replacement, reduction and refinement to minimise the use of animals, then the only model that really counts is the patient model—and here, through study design refinement, it should be possible to construct a study with the best chance of revealing a positive result when powered appropriately.
